# Spatio-temporal analysis of association between incidence of malaria and environmental predictors of malaria transmission in Nigeria

**DOI:** 10.1038/s41598-019-53814-x

**Published:** 2019-11-25

**Authors:** Oluyemi A. Okunlola, Oyetunde T. Oyeyemi

**Affiliations:** 1 Department of Mathematics, University of Medical Sciences, Ondo, Nigeria; 2 Department of Biological Sciences, University of Medical Sciences, Ondo, Nigeria; 3 Diagnosis and Therapy of Infectious Diseases and Cancer Laboratory, René Rachou Institute, Fundação Oswaldo Cruz, Belo Horizonte, Minas Gerais, Brazil

**Keywords:** Microbiology, Parasitology

## Abstract

Malaria still poses a significant threat in Nigeria despite the various efforts to abate its transmission. Certain environmental factors have been implicated to increase the risk of malaria in Nigeria and other affected countries. The study aimed to evaluate the spatial and temporal association between the incidence of malaria and some environmental risk factors in Nigeria. The study used malaria incidence and environmental risk factors data emanating from 2015 Nigeria Malaria Indicator Survey accessed from the Demographic and Health Survey database. A total of 333 and 326 clusters throughout the country were used for malaria incidence study and environmental variables respectively. The spatial autocorrelation of malaria incidence and hotspot analysis was determined by the Moran’s diagram and local Moran’s *I* index, respectively. The relationships between the malaria incidence and the ecological predictors of transmission were analysed in all the six geopolitical zones of Nigeria from 2000–2015 using ordinary least square (OLS), spatial lag model (SLM), and spatial error model (SEM). Annual rainfall, precipitation and proximity to water showed significant positive relationship with the incidence rate of malaria in the OLS model (P < 0.01), whereas aridity was negatively related to malaria incidence (P < 0.001) in the same model. The rate of incidence of malaria increased significantly with increase in temperature, aridity, rainfall and proximity to water in the SEM whereas only temperature and proximity to water have significant positive effect on malaria incidence in the SLM. The modelling of the ecological predictors of malaria transmission and spatial maps provided in this study could aid in developing framework to mitigate malaria and identify its hotspots for urgent intervention in the endemic regions.

## Introduction

Malaria is a major public health concern and has continued to be one of the major focus of Sustainable Development Goals^1^. Efforts channeled towards reducing malaria incidence have yielded some positive results as reports have shown decrease in incidence and mortality rates by 37% and 60% globally, respectively^2^. A lot is still however needed to be done to further scale down the incidence of malaria as over 200 million cases and more than 400,000 malarial-associated deaths were reported in 2017 alone^3^. Reports showed that 15 countries shared the highest burden of malaria as 80% of the deaths are concentrated in these countries^2^. Sub-Saharan African countries account for 92% of the global malarial burden^4^. Nigeria is one of the most affected countries in the world with estimated 53.7 million cases and 79,800 deaths corresponding to one-fourth and one-fifth of the overall global picture, respectively^3^.

A number of environmental and ecological factors are known to influence transmission of malaria. Chief among these are rainfall and temperature. Rainfall especially when it is heavy washes-off many of the breeding sites of mosquito vectors of malaria parasites while temperature determines the duration of development of mosquito larvae in the environment and parasite development within the vector^5,6^. Precipitation is directly related to rainfall and it is an important factor that influences the bionomics of mosquito vectors of malaria parasites^7^. Modification of environment caused by impoundment for dam construction and irrigation schemes can influence the type and distribution of mosquito breeding sites^8^.

The application of spatial analysis of risk factors including environmental factors that aid transmission is very important in the fight against several vector borne diseases including malaria. Three environmental variables including stream density, road density, and land surface have been observed to be significantly associated with West Nile Virus using least squares regression (LSR) spatial analysis^9^. Visceral leishmaniasis transmission hotspots were also identified using global and local autocorrelation analyses^10^. In Bangladesh, spatial models showed normalized difference vegetation index (NDVI) as the best leading indicator of incidence of malaria transmission. Vegetation greenness was negatively correlated with incidence of malaria^11^. Despite the burden of malaria in Nigeria, the use of spatial statistics to examine interrelationship between incidence of malaria and prevailing environmental factors are still relatively understudied. This method is very useful in identifying disease hotspots within a specific region for possible intervention.

The identification of malaria transmission hotspots through the use of spatial statistics for targeted intervention is important because if interventions are not targeted, residual malaria transmission are likely to persist in hotspots^12^. Studies in East and West African countries have supported the observations on malaria hotspots persistence following overall reduction in malaria transmission^13,14^. This can have a serious implication in malaria control as transmission hotspots may stall intervention programmes. So, a logical and viable control intervention will focus more on malaria hotspots. Given the problem of low availability of resources in many malaria endemic regions, spatial analysis for identification of hotspots for targeted control becomes more cost-effective. The study therefore attempts to investigate the spatial and temporal variation in malaria incidence rates using a nationally representative Malaria Indicator Survey of 2015 which covered 326 clusters in the six geopolitical zones of Nigeria for the period 2000–2015. It is proposed that certain environmental factors significantly influence the incidence rate of malaria in Nigeria in space and time. To establish this, a number of exploratory and spatial statistical models were used. It is expected that the models will identify malaria transmission clusters in Nigeria for necessary interventions.

## Results

### Malaria incidence in Nigeria

The incidence rate of malaria was presented in Table 1. Generally, the incidence of malaria was higher in the Northern than in the Southern region of Nigeria. The result showed that the incidence of malaria was significantly higher in the North Central region of Nigeria than the rest of the country (P < 0.05). No significant variation in incidence of malaria was observed in the three geopolitical zones of Nigeria including the South East, South South and South West (P > 0.05). Malaria incidence rate in rural residential areas (0.430 ± 0.103) was significantly higher than in urban settings (0.368 ± 0.123) (P < 0.05) (Table 2).

**Table 1:** Malarial incidence rate in the six geopolitical zones of Nigeria.

Geo-political zones	Number	Percentage	Mean malarial Incidence (±SD)
North Central	236	18.7	0.451 ± 0.095^a^
North East	192	15.2	0.423 ± 0.090^b^
North West	248	19.6	0.442 ± 0.105^ab^
South East	180	14.2	0.369 ± 0.132^c^
South South	212	16.8	0.356 ± 0.120^c^
South West	196	15.5	0.346 ± 0.130^c^
Total	1264	100.0	

Note: Mean ± SD with different superscript are significant at 5% level with a > b > ab > c.

**Table 2:** Distribution and incidence of malaria by residence type.

Residence type	Number	Percentage	Mean malarial incidence (±SD)	T-statistic	*P value*
Rural	740	58.5	0.430 ± 0.103	9.750	0.0001
Urban	524	41.5	0.368 ± 0.123		
Total	1264	100.0			

Between 2000 and 2005, there was no significant difference in the incidence rate of malaria in Nigeria. However, a significant drop in mean incidence from 0.436 ± 0.112 in 2005 to 0.377 ± 0.120 in 2010 was observed (Table 3). In 2015, a further significant decrease in mean incidence of malaria was observed (P < 0.05). The variations in mean incidence rate of malaria and environmental factors (from 2000–2015) that influence malaria incidence in the six geopolitical zones in Nigeria is presented in Table 2. The mean incidence of malaria was consistently higher from 2000 to 2005 in all the geopolitical zones of Nigeria except in the North West where the incidence was significantly lower in 2005 (0.428 ± 0.092) compared to the value reported (0.476 ± 0.113) in 2010.

**Table 3:** Variations in mean malarial incidence rate and environmental factors in Nigeria (2000–2015).

Region	Year	Malaria Incidence	Maximum Temperature (°C)	Aridity (%)	Rainfall (mm)	Precipitation (mm)
North-Central	2000	0.503 ± 0.076^a^	32.389 ± 0.973^b^	27.364 ± 5.831^a^	1267.546 ± 156.595^b^	109.973 ± 14.933^a^
	2005	0.498 ± 0.061^b^	32.571 ± 0.947^b^	26.22 ± 4.634^a^	1187.632 ± 140.722^c^	103.717 ± 15.556^b^
	2010	0.415 ± 0.109^c^	33.016 ± 0.941^a^	26.736 ± 5.985^a^	1348.328 ± 134.733^a^	107.707 ± 14.716^ab^
	2015	0.387 ± 0.069^c^	32.649 ± 0.966^b^	22.598 ± 4.743^b^	1184.479 ± 156.579^c^	91.889 ± 13.082^c^
North-East	2000	0.455 ± 0.078^a^	33.77 ± 1.565^c^	14.616 ± 7.62^ab^	887.503 ± 370.037^a^	72.482 ± 24.003^a^
	2005	0.482 ± 0.077^a^	34.526 ± 1.642^ab^	15.944 ± 5.908^a^	877.478 ± 300.362^a^	79.214 ± 17.352^a^
	2010	0.393 ± 0.087^b^	34.757 ± 1.704^a^	15.719 ± 6.507^a^	909.753 ± 342.482^a^	80.129 ± 18.389^a^
	2015	0.364 ± 0.065^b^	34.051 ± 1.584^bc^	12.572 ± 5.859^b^	882.297 ± 284.656^a^	62.516 ± 17.136^b^
North-west	2000	0.453 ± 0.115^ab^	33.831 ± 1.348^c^	11.857 ± 4.573^b^	762.318 ± 276.245^b^	64.966 ± 18.075^b^
	2005	0.428 ± 0.092^bc^	34.505 ± 1.324^ab^	14.111 ± 4.87^a^	808.942 ± 215.029^b^	77.34 ± 17.991^a^
	2010	0.476 ± 0.113^a^	34.935 ± 1.301^a^	13.599 ± 4.132^a^	909.479 ± 271.744^a^	75.53 ± 14.85^a^
	2015	0.412 ± 0.089^c^	34.13 ± 1.275^bc^	10.09 ± 3.435^c^	753.936 ± 212.556^b^	55.264 ± 12.364^c^
South-East	2000	0.448 ± 0.133^a^	31.627 ± 0.253^ab^	51.793 ± 6.446^a^	2116.939 ± 279.463^a^	165.393 ± 10.932^a^
	2005	0.447 ± 0.115^a^	31.541 ± 0.303^b^	48.366 ± 7.53^b^	1957.35 ± 344.272^b^	150.984 ± 13.98^b^
	2010	0.295 ± 0.088^b^	31.748 ± 0.294^a^	51.655 ± 9.055^a^	2182.786 ± 330.793^a^	168.111 ± 18.447^a^
	2015	0.285 ± 0.083^b^	31.721 ± 0.269^a^	44.222 ± 7.402^c^	1762.955 ± 232.558^c^	146.53 ± 15.752^b^
South-South	2000	0.449 ± 0.101^a^	31.397 ± 0.200^b^	58.175 ± 7.759^b^	2590.655 ± 592.732^ab^	172.195 ± 16.292^b^
	2005	0.388 ± 0.131^b^	31.253 ± 0.209^c^	56.564 ± 8.01^b^	2411.414 ± 505.343^b^	164.158 ± 17.919^c^
	2010	0.304 ± 0.085^c^	31.482 ± 0.258^ab^	65.096 ± 7.114^a^	2698.811 ± 610.783^a^	196.391 ± 15.62^a^
	2015	0.283 ± 0.073^c^	31.511 ± 0.236^a^	52.22 ± 8.050^c^	2131.962 ± 417.486^c^	161.662 ± 19.145^c^
South-west	2000	0.351 ± 0.154^ab^	31.687 ± 0.455^b^	33.611 ± 5.158^b^	1352.185 ± 213.771^b^	111.014 ± 14.541^b^
	2005	0.375 ± 0.128^a^	31.448 ± 0.43^c^	32.291 ± 4.516^b^	1318.17 ± 206.948^b^	101.556 ± 11.173^c^
	2010	0.349 ± 0.120^ab^	31.887 ± 0.412^a^	41.962 ± 6.087^a^	1561.039 ± 221.844^a^	135.169 ± 16.022^a^
	2015	0.311 ± 0.112^b^	31.776 ± 0.455^ab^	29.371 ± 4.003^c^	1298.87 ± 184.795^b^	98.691 ± 10.864^c^
Overall	2000	0.444 ± 0.121^a^	32.498 ± 1.397^b^	32.00 ± 18.449^b^	1468.399 ± 736.554^b^	113.937 ± 44.39^b^
	2005	0.436 ± 0.112^a^	32.705 ± 1.702^b^	31.468 ± 16.835^b^	1403.174 ± 651.458^bc^	111.353 ± 36.823^b^
	2010	0.377 ± 0.120^b^	33.044 ± 1.727^a^	34.828 ± 20.02^a^	1577.219 ± 738.002^a^	124.939 ± 47.366^a^
	2015	0.344 ± 0.097^c^	32.694 ± 1.449^b^	27.719 ± 16.544^c^	1315.455 ± 546.891^c^	100.779 ± 42.274^c^

Note: Mean ± SD with different superscript are significant at 5% level with a > b > ab > bc > c.

Generally, the Southern Nigeria recorded lower mean temperature, ranging from 31.253 °C ± 0.209 in the South South in 2005 to 31.887 °C ± 0.412 in the South West in 2010. There was no specific pattern in mean temperature variation in the six regions in Nigeria. Aridity increased generally in the Northern part of Nigeria from 2005–2015 but a significant increase in aridity from 2010 to 2015 was particularly common in all the Northern regions (Table 3). The South South with aridity index 58.175 ± 7.759 in 2000 and 52.22 ± 8.050 in 2015 was the most humid. There was direct relationship between aridity and decrease in rainfall. The Northern regions with increase aridity also recorded lower mean rainfall. There was no significant difference in the mean rainfall patterns in the North East region between 2000 and 2015. The South South region with the highest mean rainfall (2698.811 mm ± 610.783) in 2010 also recorded the highest mean precipitation (196.391 mm ± 15.62).

### Non-spatial relationships between incidence of malaria and environmental variables

A significant positive correlation occurred between the incidence rate of malaria and maximum temperature (r = 0.094, P < 0.05), and proximity to water (r = 0.216, P < 0.01). A significant negative correlation, however, was recorded between malaria incidence rate and aridity (r = −0.133, P < 0.01), rainfall (r = −0.094, P < 0.05) and precipitation (r = −0.100, P < 0.05). Rainfall correlated negatively with maximum temperature (r = −0.791, P < 0.001).

### Non-spatial versus spatial regression on impact of environmental variables on incidence rate of malaria

The predictors such as annual rainfall, precipitation and proximity to water have positive and significant effect on the incidence rate of malaria (P < 0.01) in the OLS model whereas aridity was negatively related to malaria incidence rate (P < 0.001) in the same model. The rate of incidence of malaria increased significantly with increase in temperature in the SLM and SEM spatial models (Table 4). In addition to negative and significant impact of precipitation on incidence of malaria in SEM, the coefficient of temperature, aridity and proximity to water were also positive and significant. The SEM model with smallest information criteria value (AIC = −2686.945, BIC = −2645.809) provide best explanation on impact of selected environmental factors on malaria incidence. The non-spatial OLS perform poorly compared to the spatial models.

**Table 4:** Spatial models showing correlation between incidence rate of malaria and environmental variables.

	Ordinary Least Square	Spatial Lag Model	Spatial Error Model
Max. Temperature	0.070	0.250**	0.599***
Aridity	−0.114***	0.025	0.734***
Annual rainfall	0.068**	0.010	0.045
Precipitation	0.131**	0.002	−0.697***
Proximity to water	0.029***	0.014***	0.016***
Intercept	−0.889	−1.149**	−1.399**
Rho		0.886***	
Lambda			0.981***
Sigma		0.084***	0.079***
R-square	0.107	0.473	0.528
AIC	−1996.753	−2586.866	−2686.945
BIC	−1965.900	−2545.730	−2645.809

### Moran’s *I* statistics for determination of spatial autocorrelation

**Figure 1:**
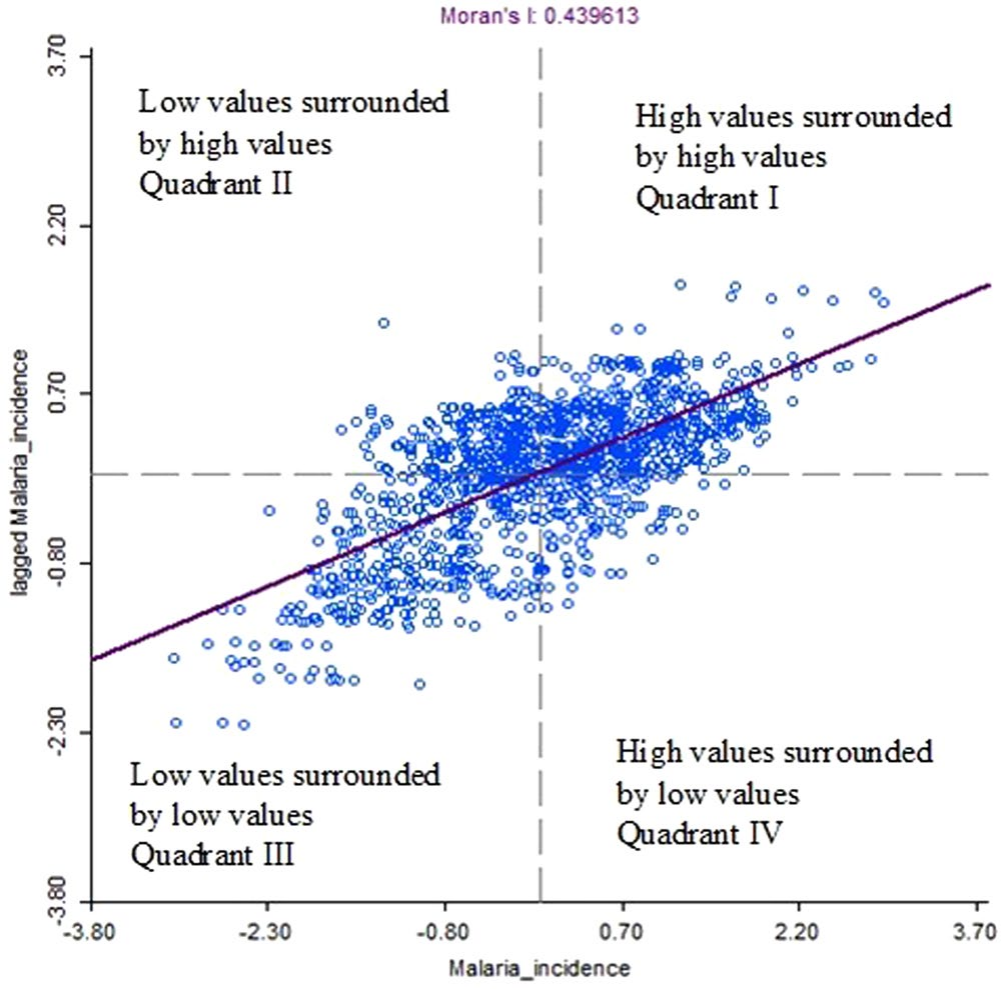
Mean Moran’s *I* values for local spatial autocorrelation for malaria incidence at varying spatial lags.

**Figure 2:**
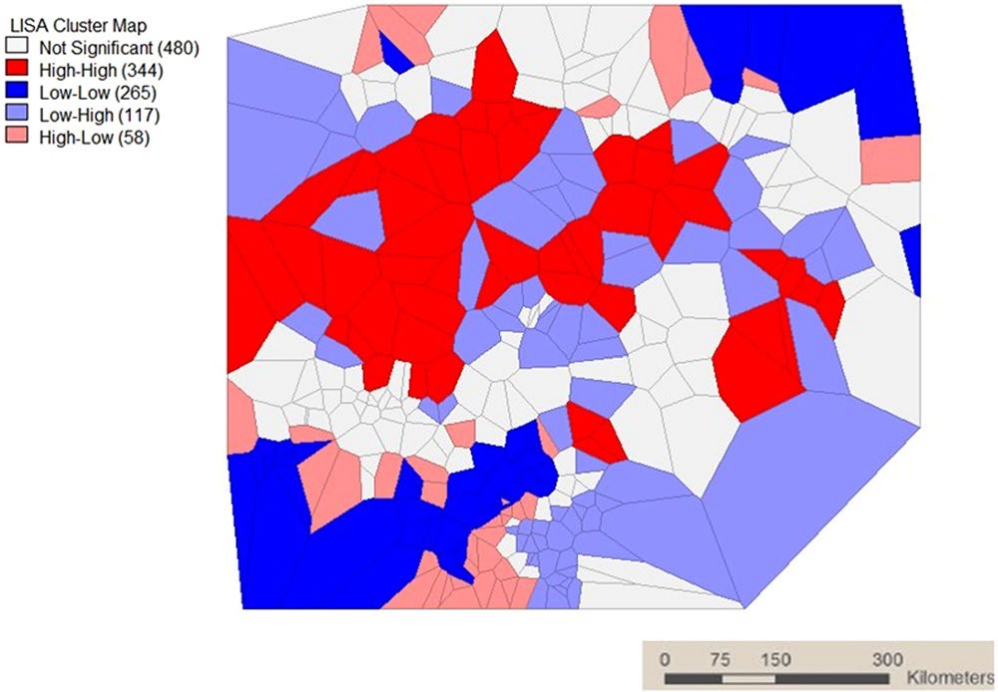
LISA cluster map.

A significant Moran’s *I* statistics of 0.440 was observed (P < 0.05) Fig. 1. Figure 2 showed the Moran’s *I* scatter plot of incidence rate of malaria. Points in quadrant I showed clusters with high malaria incidence rate (relative to average of the 344 clusters) was surrounded by clusters of high malaria incidence rate (HH), quadrant II showed regions with low malaria incidence rate surrounded by clusters with high malaria incidence rate (LH), quadrant III showed regions with low malaria incidence rate surrounded by clusters of low incidence rate of malaria (LL), and quadrant IV showed regions with high incidence rate of malaria surrounded by clusters of low incidence rate of malaria (HL). The plots showed more cluster points in quadrant I and III.

**Figure 3:**
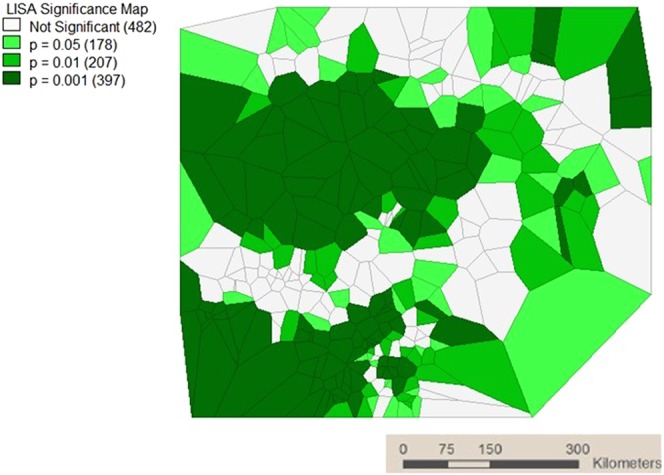
LISA significance map.

LISA significance map of Local Moran’s *I* test for local spatial autocorrelation patterns of incidence rate of malaria was presented in Fig. 3. The bright green and green shade clusters represented regions of malaria incidence rate which showed significant local spatial autocorrelation (P < 0.05).

### Spatio-temporal distribution of malaria

**Figure 4:**
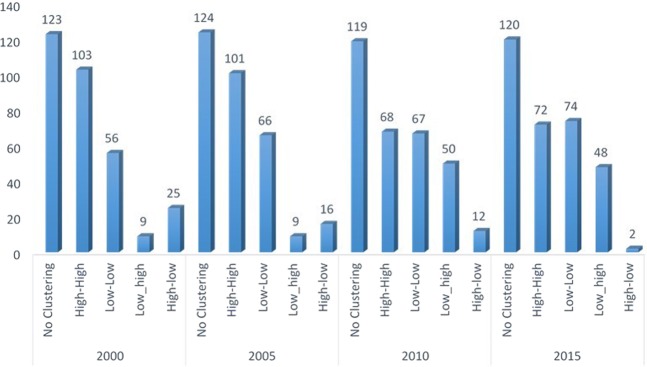
Spatial-temporal distribution of malaria.

**Figure 5:**
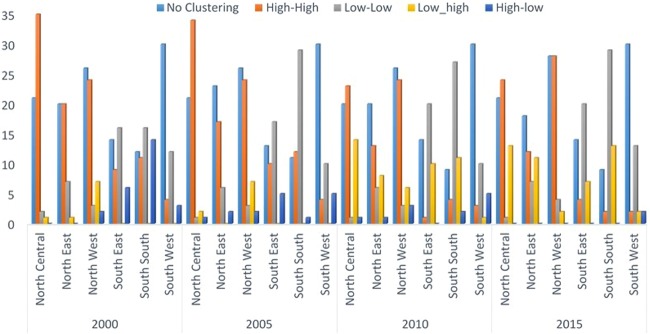
Spatial-temporal distribution of incidence of malaria by geopolitical zones.

The spatio-temporal distribution of malaria is presented in Fig. 4. Further analysis of LISA showed that there were 344 hotspots locations distributed across the six geopolitical zones. The distribution of these hotspots locations revealed that the Northern geopolitical zones have larger proportion with North Central, North West and North East having 33.7%, 29.1% and 18.0%, respectively. Whereas, the proportion of hotspots in the South South, South East, and South West were 8.4%, 7.0% and 3.0%, respectively (Table 5). The distribution of hotspots of malaria incidence depicted in Fig. 5 showed similar pattern across the geopolitical zones over time with the North Central taking the lead and immediately followed by the North West. However, there was a systematic decrease in the number of hotspots clusters in each of the geo-political zone from year to year. In general, the hotspots clusters reduced by 51.5% between 2000 and 2010.

**Table 5:** Analysis of local indicator of spatial autocorrelation (LISA) by geopolitical Zones.

Region	No Clustering (%)	High-High (%)	Low-Low (%)	Low-high (%)	High-low (%)	Total (%)
North Central	83 (17.1)	116 (33.7)	5 (1.9)	30 (25.9)	2 (3.6)	236 (18.7)
North East	81 (16.7)	62 (18.0)	26 (9.9)	20 (17.2)	3 (5.5)	192 (15.2)
North West	106 (21.8)	100 (29.1)	13 (4.9)	22 (19.0)	7 (12.7)	248 (19.6)
South East	55 (11.3)	24 (7.0)	73 (27.8)	17 (14.7)	11 (20.0)	180 (14.2)
South South	41 (8.4)	29 (8.4)	101 (38.4)	24 (20.7)	17 (30.9)	212 (16.8)
South West	120 (24.7)	13 (3.8)	45 (17.1)	3 (2.6)	15 (27.3)	196 (15.5)
Total	486 (100)	344 (100)	263 (100)	116 (100)	55 (100)	1264 (100)

## Discussion

Malaria continues to be a serious threat in all regions of Nigeria. Studies across Nigeria have attributed higher prevalence of malaria as high as 70–99% to the South^15–17^. The reasons in support of this are the higher rainfall patterns, more water bodies and heavy forest which are predominant environmental factors that characterise the South, and which aid malaria transmission in the region^18^. However, this study showed that the incidence of malaria which is the number of new malaria cases during 2000–2015 period of time was significantly higher in the Northern than in the Southern Nigeria. The North East Nigeria although recorded higher incidence than all the Southern regions, the incidence in the region was lower than the two other geopolitical zones in the North. This lower incidence could be attributed to larger coverage of insecticide treated nets (ITNs) in the region compared to the rest. In a report by the Nigeria Malaria Indicator Survey in 2010, 67.4% of individuals from the North East claimed ownership of at least one ITN, while 32.7% and 59.7% were reported for North Central and North West respectively^19^.

Although the prevailing climatic conditions appeared to be negatively correlated with malaria transmission in Northern Nigeria, areas surrounding the confluence of the Rivers Niger and Benue in the North Central and many isolated areas of the North East and North West parts of Nigeria have been reported to have as high as 70% prevalence of malaria^18^. Poor access to health care and public health services in isolated areas of the North and impact of Rivers Niger and Benue could be responsible for such high endemicity of malaria in the regions. Higher malaria incidence in the rural areas of Nigeria could be attributed to the prevailing cultural practices in the areas that could predisposed the people to infection by malaria parasites. Many of the rural areas both in the North and South are isolated and very difficult to access. Besides, poor socio-cultural development of the areas and lack of basic social amenities often discourage health workers posted to the places. Because of these fundamental problems, health service delivery in those areas is often poor and it usually undermines the people’s access to good health care services. Although malaria incidence in urban centers is lower in this study, there is however, stable transmission of malaria in Nigerian urban regions. One important reason is that some urban centers in Southern Nigeria are located in the coastal regions thus providing suitable breeding sites for mosquito vectors of malaria parasites. Urban agricultural development involving irrigation is common in the North and this could facilitate malaria transmission in the region. Poor drainage systems and creation of artificial vector breeding sites like ditches and tyre tracks during heavy downpour are common in Nigerian urban centers.

The Roll Back Malaria (RBM) Partnership initiated in 2000 seemed not to have yielded any significant positive result as malaria incidence in some of the regions in Nigeria either remained the same or rose significantly between 2000 and 2005. The failure of RBM Partnership necessitated the development of the Global Malaria Action Plan in 2008. It appeared that the new initiative yielded some positive results in Nigeria as there were significant reduction in the incidence of malaria between 2010 and 2015 in all the six geopolitical zones of Nigeria. This is further supported by the overall significant decrease in incidence of malaria between 2010 and 2015.

Malaria is a disease whose transmission is greatly influenced by environmental factors. These factors are good predictors of transmission but could share non-linear relationship with mosquito abundance and malaria transmission^18^. The impact of environmental variables on malaria transmission can be adequately established by spatial statistical models which can predict the transmission of malaria both in space and also in time. Rise in temperature shortens the blood meals-seeking behaviour of female *Anopheles* mosquito, therefore causing a corresponding decrease in ovulation and production of juvenile mosquitoes. The temperature of as high as 34 °C which is the average upper limit temperature recorded in the Northern Nigeria has been reported to cause reduction in the gonotrophic cycle length of mosquitoes^20^. The general temperature range (27–29 °C) in many areas of Nigeria falls within the optimum value for the development of sporozoites within the mosquitoes^21^. The daily survival of mosquitoes is also influenced by temperature. Mosquitoes’ daily survival rate of about 90% has been attributed to temperatures between 16 °C and 36 °C^21^. Whichever way, it is clear that temperature is a very important factor that aids transmission of malaria both in the Northern and the Southern parts of Nigeria. This claim was supported by a positive correlation between incidence of malaria and temperature. More importantly, the very significant relationships the SLM and SEM spatial models showed with temperature makes the later a significant predictor of malaria transmission in Nigeria. In fact, the positive and significant spatial lag coefficient in the SLM indicates that malaria incidence rate in one cluster depends directly on the rate of incidence in its neighboring clusters affected by temperature.

Rainfall and precipitation are also two factors that affect incidence of malaria in Nigeria. However, because of the variation in the rainfall patterns of the Northern and Southern parts of Nigeria, these may affect malaria transmission dynamics differently. The Southern regions enjoy a longer duration of rainfall than the North. So, transmission is usually higher at the onset of rainy season and the beginning of dry season^22^. The characteristic rainfall patterns in the Southern Nigeria create shallow water pockets suitable for breeding of *Anopheles gambiae* which is the main mosquito vector of malaria parasite in Nigeria^23^. The negative correlation in the non-spatial statistical analysis is suggestive of a negative impact continuous heavy rainfall especially during the peak rainy season may have on malaria parasites vectors and eventual transmission of the disease. This could explain the reason the incidence of malaria is relatively lower in the region compared to the Northern part of Nigeria. Precipitation has been considered to be the most important climatic factor that influence incidence of malaria in the lowlands^24^. Our study showed that precipitation is strongly correlated with rainfall. The impact of precipitation in malaria transmission is both direct and indirect especially where dams are situated. It raises reservoir’s water level and creates potential mosquito breeding sites along the shorelines^24^. Previous findings from Nepal using generalized additive mixed models (GAMM) however, showed that maximum temperature and rainfall were not significantly associated with malaria incidence^25^. The same was observed with rainfall in Bangladesh^11^ but our study was similar to reports from India and Sri Lankan which reported negative correlation between rainfall and incidence of malaria^26,27^. The difference in the sign and significance of the parameters between the OLS and the spatial models confirms the assertion that OLS models remain unbiased in the presence of spatial autocorrelation but remain inefficient and inconsistent in SEM and SLM models, respectively. Misleading conclusion is inevitable when OLS technique is used in analysing sample data collected for regions or points I space. The significance of the spatial autoregressive parameter ρ (Rho) in the SLM and λ (Lambda) in the SEM indicated that spatial autocorrelation exists in the data and that the spatial model is more appropriate than standard aspatial model which is prone to misleading result and under or over estimation of the parameters. This result agreed with Anselin^28^ and LeSage and Kelly^29^ that OLS result is inconsistent and inefficient in the SLM and SEM models, respectively

Aridity is higher in the North than in the South and its increase influences malaria transmission by reducing mosquito biting rate and the adult lifespan than the extrinsic incubation period for malaria parasite^24,30^. Using the work of De Martonne^31^, the North East and North West zones of Nigeria are semi-arid, while the North Central is semi-humid. The South West, South East and South South regions of Nigeria are humid, very humid, and extremely humid respectively.

A significant Moran’s *I* statistics also denotes the same and justifies that malaria incidences in the nearby clusters are more related than those far away. The univariate Moran’s scattered plots showed more points in quadrant I and III denoting a positive spatial autocorrelation pattern in incidence of malaria among clusters in different regions of Nigeria. The extent of this autocorrelation was tested by LISA model which is a class of spatial statistics that provides information specific to clusters and estimates the extent of spatial autocorrelation of malaria incidence in a particular cluster in relation to its neighbours. The over 700 clusters with significant local spatial autocorrelation patterns in incidence of malaria as revealed by the LISA significance plots shows that there are indeed presence of spatial association in incidence of malaria in Nigeria. The reduction in malaria hotspot clusters from 2000–2015 indicated that the various interventions from government and international agencies to combat malaria in the country has been productive.

One limitation of spatial modelling is that while infectious disease data has a lot of intra- and inter-annual variability depending on epidemic and non-epidemic periods, the regression analysis assumes the association between exposure and outcome to be stationary over time^32^.

## Conclusion

Our study has shown that malaria is still a serious problem in all the regions of Nigeria with environmental factors like rainfall, temperature and aridity playing important roles in transmission of the disease. There is more malaria incidence in the North than in the South and rural than urban areas. The spatial statistical models adopted are important to design a prompt and early malaria transmission mitigation support system in suspected regions. The models can help to generate malaria risk map and spatially channel available resources to the disease hot spots.

## Materials and Methods

### Study area

The study was carried out in Nigeria, a country in sub-Saharan African region, located between latitudes 4°16′ and 13°53′ North and longitudes 2°40′ and 14°41′ East. The country has a total surface area of approximately 923,768 square kilometers and density of 212.04 individuals per square kilometers. One of the country most severe public health problems is malaria and the climatic conditions of the country make it suitable for recurrent malaria transmission. There have been various interventions from government and international agencies to mitigate the burden of this tropical disease.

### Data source

The Demographic and Health Survey Programme (DHSP) assists countries worldwide in the collection and use of data to monitor and evaluate population, health, and nutrition programmes. Data emanating from the survey are processed and made available upon request for download through the Demographic and Health Surveys (DHS) Programme website. The data often come with geospatial covariates and it is often difficult to link these covariates with the DHS Programme’s data to determine the impact of location on health outcomes. To alleviate the difficulty, the DHS Programme Geospatial Team developed a set of standardised files of the most commonly used geospatial covariates already linked with the dataset.

The covariate variables came from two types of data: raster and vector. Raster data, such as images and modeled surfaces, rely on pixels or cells to convey their data values. On the other hand, vector data, such as points, lines, and polygons, show the discrete location or boundary of a feature. Because of the differences in the data types, the methods needed to extract meaningful values varied. Firstly, Geospatial covariate layers (i.e. modeled surfaces) that are relevant to the DHS Programme indicators were acquired from Digital Globe (~35 cm resolution) remotely sensed imagery. GPS coordinates representing the location of a survey cluster were obtained from the DHS programme. In addition to modeled surfaces, vector (polygon and line) data, which were obtained from various publicly available sources were also included. Secondly, Raster and vector datasets were imported and linked to GPS using a standalone Python programming language script and ArcGIS, respectively.

The study used data emanating from 2015 Nigeria Malaria Indicator Survey (NMIS) accessed at the DHS website. The 2015 Nigeria Malaria Indicator Survey was implemented by the National Malaria Elimination Programme (NMEP), the National Population Commission (NPC), and the National Bureau of Statistics (NBS) and other international agencies from October 2015 through November 2015. The International Classification of Functioning, Disability and Health provided technical assistance as well as funding to the project through the DHS Programme; a project funded by the United States Agency for International Development (USAID)^19^.

Rainfall data was obtained from a satellite-based rainfall product called the Climate Hazards Group InfraRed Precipitation with Stations (CHIRPS) which has high temporal and spatial resolution^33^. Maximum temperature and precipitation data were obtained from the Climate Research Unit (CRU) of the University of East Anglia, UK, which produces a range of global climate time series gridded data, derived from meteorological stations across the world’s land areas. The datasets were provided on high resolution (0.5 × 0.5 degrees) grids over the period 1901–2016^34,35^. Aridity was modeled using data available from the WorldClim Global Climate Data and was updated for the period of 2000, 2005, 2010 and 2015 using high resolution grids obtained from the CRU datasets^35^. Proximity to water data was extracted from lakes dataset (L2) at full resolution and the shoreline dataset (L1), also at full resolution, in the Global Self-consistent, Hierarchical, High-resolution Shoreline (GSHHG) database. The datasets used were based on the World Vector Shorelines, CIA World Data Bank II, and Atlas of the Cryosphere^36,37^.

### Sampling procedures

A two-stage sampling strategy was adopted for the 2015 NMIS. In the first stage, nine clusters (EAs) were selected from each state, including the Federal Capital Territory (FCT). The sample selection was done in such a way that it was representative of each state. The result was a total of 333 clusters throughout the country, 138 in urban areas and 195 in rural areas. The geospatial covariates of 2015 NMIS housed data on malaria incidence (defined as the average number of people per year who show clinical symptoms of *Plasmodium falciparum* malaria within the 2 km (urban) or 10 km (rural) buffer surrounding the DHS survey cluster location) as well as the environmental variables measured using remote sensing within the 2 km (urban) or 10 km (rural) buffer surrounding the DHS survey cluster location for 326 clusters within the country over interval of five years (2000, 2005, 2010 and 2015) were used. To ensure completeness of the dataset, all empty cells and inconsistent cases were removed and the retained samples became 1264 as against original cases of 1304 which amount to 96.9% of the total cases. The distribution of the retained samples by geopolitical zones and residence type are shown in Tables 1 and 2.

### Statistical and spatial analyses

Descriptive statistical analysis, mean difference and association between malaria incidence and the environmental variables were done using frequency counts, percentages, independent t-test, Pearson’s Product Moment Correlation (PPMC) and one-factor analysis of variance (ANOVA).

The main motivation for applying spatial statistical model is the existence of spatial autocorrelation. This is analogous to time series serial autocorrelation except that it is multidirectional while serial autocorrelation is unidirectional. Global spatial autocorrelation is commonly detected in georeferenced data by the Moran’s *I* test-statistics^28^ and it is given as;

1 $$I=\frac{n}{{S}_{o}}\frac{{\sum }_{i=1}^{n}{\sum }_{j=1}^{n}{w}_{ij}({x}_{i}-\bar{x})({x}_{j}-\bar{x})}{{\sum }_{i=1}^{n}{({x}_{i}-\bar{x})}^{2}}\,{\rm{or}}\,{\rm{in}}\,{\rm{matrix}}\,{\rm{form}}\,{\rm{as}}\,I=\frac{n}{{S}_{o}}\frac{x^{\prime} Wx}{x^{\prime} x}$$

Where n is the *n* × 1 vector of a random variable which has been standardised such that the mean and variance are 0 and 1, respectively. W is an *n* × *n* row standardised (row sum equal to 1) spatial weight matrix and *S*_*o*_ is the sum of the elements of *W*. *W* captures the nature of connected among the spatial units in the data and this can be conceived in the topological notion of neighbourhood. In this study, Queen Contiguity criterion is adopted, which stipulates that two areas are neighbours when they share a common side or vertex. A first order queen contiguity matrix is defined as ${W}_{ij}=1$ if clusters *i* and *j* share common side or vertex and zero if otherwise. The diagonal element of *W* is constraint to be zero so as to prevent a cluster from being a neighbour to itself. Torres-Preciado *et al*.^38^ reported that such matrix facilitates the interpretation of neighbourhood phenomenon underlying the administrative breakdown and improves the efficiency algorithms during the estimation process. Moran’s *I* index takes value between −1 and 1 and it can be interpreted as a product moment correlation coefficient. The positive values of Moran’s *I* indicate that observation of similar values occurs as neighbour whereas negative values signify that both high and low value observations occur as neighbours. A Moran’s *I* value of zero signifies a random spatial distribution. A local indicator of spatial autocorrelation (LISA)^34^ or the so-called local Moran’s *I*, test for local spatial autocorrelation. The LISA indicates significant spatial clustering and sums up proportional to the global Moran’s *I*^39^. It is possible for the dataset to have significant local spatial clustering but no global spatial autocorrelation.

Based on the likelihood that malaria incidence in a given cluster might be influenced by the similar incidence in a nearby cluster, Moran’s diagram was employed to have a rapid and global knowledge of the global spatial autocorrelation in malaria incidence while LISA was used to detect the hot and cold spots clustering location in the sample. As earlier described, the positive value of Moran’s *I* will be interpreted as high values of malaria incidence and are grouped together in space whereas its negative signified that the dissimilar values of malaria incidence come together geographically. If it is zero, then spatial dependence is absent in the variable and in this case the assumption of independence holds. The cluster and significant map showed the hot and cold spot locations.

Prior to the production of the diagram and map, connectivity matrix among the clusters was created using the coordinates of the cluster displaced by up to 2 kilometers (for Urban points) and 10 kilometers (for Rural points)^40^ based on the first order queen contiguity criterion earlier illustrated. In the diagram, the values of malaria incidence on the *x*-*axis* was plotted against the average values of the malaria incidence for the neighbouring observations Wy (lagged malaria incidence) in the *y*-*axis*. The diagram has four quadrants as shown in Fig. 1. The value above the diagram is the global Moran’s *I* index. If the value is close to zero, it means malaria distribution is spatially random, while a positive value indicates spatial clustering^41^.

Due to the spatial nature of the data and the possibility that malaria incidence in one location may be influenced by similar values in another location, three regression specifications were used to model the relationship between incidence of malaria and environmental factors. The non-spatial regression, Spatial Lag Model (SLM), and Spatial Error Model (SEM) as shown in Eqs 2–4. Ordinary Least Square (OLS) estimation method was used for equation while Eqs 3 and 4 were estimated by maximum likelihood method because OLS estimation of Eq. 3 has been reported to be inconsistent^42,43^ while in the case of Eq. 4, it remained unbiased but inefficient^29^.

2 $$OLS;y=X\beta +\varepsilon $$

3 $$SLM;y=\rho Wy+X\beta +\varepsilon $$

4 $$SEM;y=X\beta +\varepsilon ;\varepsilon =\lambda W\varepsilon +u$$

The OLS model is aspatial and it behaves well under the assumptions of independence of observations and homoskedastic error terms. Sample data collected for regions or points in space are not independent, but rather spatially dependent^44^. Firstly, data records at proximal locations appear to be either positively or negatively correlated, which is called spatial dependence. Secondly, in spatial data setting the homoskedastic assumption cannot hold due to lack of structural stability across space such as varying parameters or functional forms. Due violation of classical statistics assumption regarding independence and of observation and homoskedastic error terms the need for models that can account for spatial structure in their specification is necessitated. The most common way of adjusting model 2 to accommodate spatial structure is to add spatial lag of the dependent variable or the disturbance term to the model. Models 3 and 4 are spatial regression models in that the spatial lag of the dependent variable (*Wy*) and that of the disturbance term (*Wε*) have been added to their specification. The two models revert to aspatial model (model 1) when the spatial effect parameters (*ρ* and *λ*) are equal to zero.

Maximum likelihood estimation technique was derived and suggested for SLM and SEM models^28,45,46^. In this approach, the probability of the joint distribution (likelihood) of all observations is maximized with respect to a number of relevant parameters. If the regularity conditions for the log-likelihood functions are satisfied, the obtained ML estimation will achieve the desirable properties of consistency, asymptotic efficiency, and asymptotic normality. Moreover, in most situations, the resulting estimates for the regular parameters of the models are also unbiased^28^.

## Data Availability

Data will be made available on request.

## References

[1] Sewe MO, Tozan Y, Ahlm C, Rocklöv J (2017). 2017. Using remote sensing environmental data to forecast malaria incidence at a rural district hospital in Western Kenya. Sci. Rep..

[2] World Health Organisation. World Malaria Report (2015).

[3] World Health Organisation. World Health Organisation Fact Sheets, https://www.who.int/news-room/fact-sheets/detail/malaria (2019)

[4] World Health Organisation. World Malaria Report 2018. Geneva: World Health Organization (2018).

[5] Patz JA, Olson SH (2006). Malaria risk and temperature: influences from global climate change and local land use practices. Proc. Natl. Acad. Sci. USA.

[6] Stern DI (2011). Temperature and malaria trends in highland East Africa. PLoS One.

[7] Lindblade KA, Walker ED, Wilson ML (2000). Early warning of malaria epidemics in African highlands using Anopheles (Diptera: Culicidae) indoor resting density. J. Med. Ent..

[8] Keiser J (2005). Effect of irrigation and large dams on the burden of malaria on a global and regional scale. Am. J. Trop. Med. Hyg..

[9] Kala AK, Tiwari C, Mikler AR, Atkinson SF (2017). 2017. A comparison of least squares regression and geographically weighted regression modeling of West Nile virus risk based on environmental parameters. Peer J..

[10] Dewan A, Abdullah AYM, Shogib MRI, Karim R, Rahman MM (2017). Exploring spatial and temporal patterns of visceral leishmaniasis in endemic areas of Bangladesh. Trop. Med. Health.

[11] Haque (2010). The Role of Climate Variability in the spread of malaria in Bangladeshi highlands. PLoS ONE.

[12] Bousema T (2012). Hitting Hotspots: Spatial Targeting of Malaria for Control and Elimination. PLoS Med..

[13] Ernst KC, Adoka SO, Kowuor DO, Wilson ML, John CC (2006). Malaria hotspot areas in a highland Kenya site are consistent in epidemic and non-epidemic years and are associated with ecological factors. Malar J..

[14] Gaudart J (2006). Space-time clustering of childhood malaria at the household level: a dynamic cohort in a Mali village. BMC Publ. Health.

[15] Ibekwe AC (2009). Comparative prevalence level of *Plasmodium* in freshmen (first year students) of Nnamdi Azikwe University in Awka, South-Eastern, Nigeria. Malays J. Microbiol..

[16] Okonko IO (2009). Prevalence of malaria plasmodium in Abeokuta, Nigeria. Malays J Microbiol..

[17] Gunn JK (2015). Population-based prevalence of malaria among pregnant women in Enugu State, Nigeria: the healthy beginning initiative. Malar J..

[18] Onyiri N (2015). Estimating malaria burden in Nigeria: a geostatistical modelling approach. Geospat. Health.

[19] Nigeria Malaria Indicator Survey, 2010. Abuja: National Population Commission (NPC), National Malaria Control Programme (NMCP), and ICF International, pp43–58 (2010).

[20] Shapiro LLM, Whitehead SA, Thomas MB (2017). Quantifying the effects of temperature on mosquito and parasite traits that determine the transmission potential of human malaria. PLoS Biol..

[21] Craig MH, Snow RW, Sueur D (1999). A climate-based distribution model of malaria transmission in sub-Saharan Africa. Parasitol. Today.

[22] Okeke OP, Imakwu CA, Eyo JE, Okafor FC (2016). Prevalence of malaria infection in children in Anambra State, Nigeria after change of policy from presumptive/clinical to confirmed diagnosis. Animal Res. Inter..

[23] Adigun AB, Gajere EN, Oresanya O, Vounatsou P (2015). Malaria risk in Nigeria: Bayesian geostatistical modelling of 2010 malaria indicator survey data. Malar. J..

[24] Kibret S, Wilson GG, Ryder D, Tekie H, Petros B (2019). Environmental and meteorological factors linked to malaria transmission around large dams at three ecological settings in Ethiopia. Malar J..

[25] Dhimal M (2014). Spatio-temporal distribution of malaria and its association with climatic factors and vector-control interventions in two high-risk districts of Nepal. Malar. J..

[26] Bhattacharya S, Sharma C, Dhiman R, Mitra A, Climate change (2006). and malaria in India. Curr. Sci..

[27] Briet J, Vounatsou P, Gunawardena D, Galappaththy N, Amerasinghe P (2008). Temporal correlation between malaria and rainfall in Sri Lanka. Malar J..

[28] Anselin, L. Spatial Econometrics: methods and models, Kluver Academic, Dordrecht (1988).

[29] LeSage, J. P. & Kelly, P. R. Introduction to spatial econometrics. Boca Raton, FL: CRC Press (2009).

[30] Shililu J (2003). High seasonal variation in entomologic inoculation rates in Eritrea, a semi-arid region of unstable malaria in Africa. Am. J. Trop. Med. Hyg..

[31] De Martonne, E. Traité de Géographie Physique. Quatrième édition. A. Colin, Paris (1925).

[32] Cazelles B, Chavez M, McMichael AJ, Hales S (2005). Nonstationary influence of El Niño on the synchronous dengue epidemics in Thailand. PLoS Med..

[33] Climate Hazards Group. Climate Hazards Group InfraRed Precipitation with Station data 2.0, http://chg.geog.ucsb.edu/data/chirps/index.html (2017).

[34] Climate Research Unit. CRU TS v. 4.01, http://doi.org/10/gcmcz3 (2017).

[35] Harris IPD, Jones TJ, Osborn DL (2014). Updated high-resolution grids of monthly climatic observations - the CRU TS3.10 Dataset. Inter. J. Climatol..

[36] Wessel P, Walter SA (1996). Global Self-consistent, Hierarchical, High-resolution Shoreline Database. J. Geophys. Res..

[37] Wessel, P. & Walter, S. A Global Self-consistent, Hierarchical, High-resolution Geography Database Version 2.3.7, http://www.soest.hawaii.edu/pwessel/gshhg (2017).

[38] Torres-Preciado VH, Polanco-Gaytán M, Tinoco-Zermeño MA (2014). Technological innovation and regional economic growth in Mexico: a spatial perspective. Ann. Reg. Sci..

[39] Anselin L (1995). Local indicators of spatial association-LISA. Geog. Analysis.

[40] Benjamin, M., Fish, T. D., Eitelberg, D. & Dontamsetti, T. The DHS Program Geospatial Covariate Datasets Manual (Second Edition). Rockville, Maryland, USA: ICF (2018).

[41] Robertson C, Nelson TA (2014). An overview of spatial analysis of emerging infectious diseases. Prof. Geogr..

[42] Luc, A. & Bera, A. K. Spatial dependence in linear regression models with an introduction to spatial econometrics.” Statistics Textbooks and Monographs 155. Marcel Dekker A.G. 237–90 (1998).

[43] Lee LF (2002). Consistency and efficiency of least squares estimation for mixed regressive, spatial autoregressive models. Economet. Theor..

[44] Olubusoye OE, Okunlola OA, Korter GO (2015). Estimating bias of omitting spatial effect in spatial autoregressive (SAR) model. Inter. J. Stat. Appl..

[45] Anselin, L. Spatial regression. In: Fothering-ham, A. S., Rogerson, P. A. (eds). The SAGE handbook of spatial analysis. SAGE, Los Angeles, 255–275 (2009).

[46] Griffith DA (1993). Exploring relationships between semi-variogram and spatial autoregressive models. Papers Region Sci..

